# Bibliography

**Published:** 1906-02-15

**Authors:** 


### BIBLIOGRAPHY.

ORTHODONTIA. By Prof. S. H. Guilford is a new and fourth edition of this valuable little work on this popular subject, by one who has worked at it and taught it for twenty-five years to hundreds of students. Although this is a new edition of a former work, it bears evidences of many changes and the introduction of many of the newer and more practicable methods of correcting mal-occlusion of the teeth. The author has devised some new terms for expressing his ideas of conditions associated with the science and technics of orthodontia; such as ben-occlusion to substitute normal occlusion, and ant-occlusion and post-occlusion in place of mesial and distal occlusion, etc. We note with pleasure in the chapter on occlusion that the author states with emphasis that he believes in preserving the full complement of teeth and in their exact occlusal relations. This is the most important and fundamental principle of modern orthodontia, and we note also that the acceptance of this principle has made a change in the illustrations of models of cases. The models are presented of both the upper and lower, and are made with more care. In the chapter on technical manipulations we notice that the author gives modeling compound a second place to plaster of paris. We don't think modeling compound can ever be used to secure an accurate model of the teeth when in mal-occlusion. Plaster is so much more definite and about as easily manipulated and it should always be used for impressions.

There is a chapter on the construction of appliances that is good but might have been much abbreviated, since there is little occasion now for the dentist to spend his time in the manufacture of appliances which can seldom be made with economy or with the necessary exactness to be had in the ready-made appliances now to be had in all dental supply houses. The various manufactured appliances are illustrated and discussed. The chapter on retention is altogether inadequate as this is the most important feature of this work. Almost a novice who understands the principles of mechanics can move teeth, but it takes a wise and long head to devise a retention that will prove effective. None of our text books give the subject proper consideration. The author classifies mal-occlusion into two divisions:

one for all simple irregularities and another for the complex irregularities. Under the first division he names four classes and under the second he names seven classes. The difficulty with such a classification is that it is not definite and does not make it possible to speak of the various mal-positions of teeth without illustrations or models. We regard the Angle classification as much simpler and more definite. It is surprising how completely the author has covered the various methods. This shows that he keeps in touch with the great progress made in this subject in recent years. It is a good book, but we experience a sense of incompleteness because most all of the statements are so brief. The book is printed in good style by T. C. Davis & Son, Philadelphia.

